# Detection of Osmotic Shock-Induced Extracellular Nucleotide Release with a Genetically Encoded Fluorescent Sensor of ADP and ATP

**DOI:** 10.3390/s19153253

**Published:** 2019-07-24

**Authors:** Keelan J. Trull, Piper Miller, Kiet Tat, S. Ashley Varney, Jason M. Conley, Mathew Tantama

**Affiliations:** 1 Department of Chemistry, Purdue University, 560 Oval Drive, West Lafayette, IN 47907, USA; 2 Department of Pediatrics, Indiana University School of Medicine, Indianapolis, IN 46202, USA; 3 Purdue Institute for Integrative Neuroscience, Hall for Discovery Learning #399, 207 South Martin Jischke Drive, West Lafayette, IN 47907, USA; 4 Department of Chemistry, Wellesley College, 106 Central Street, Wellesley, MA 02481, USA

**Keywords:** extracellular ADP, purinergic signaling, fluorescent sensor, FRET, genetically encoded

## Abstract

Purinergic signals, such as extracellular adenosine triphosphate (ATP) and adenosine diphosphate (ADP), mediate intercellular communication and stress responses throughout mammalian tissues, but the dynamics of their release and clearance are still not well understood. Although physiochemical methods provide important insight into physiology, genetically encoded optical sensors have proven particularly powerful in the quantification of signaling in live specimens. Indeed, genetically encoded luminescent and fluorescent sensors provide new insights into ATP-mediated purinergic signaling. However, new tools to detect extracellular ADP are still required. To this end, in this study, we use protein engineering to generate a new genetically encoded sensor that employs a high-affinity bacterial ADP-binding protein and reports a change in occupancy with a change in the Förster-type resonance energy transfer (FRET) between cyan and yellow fluorescent proteins. We characterize the sensor in both protein solution studies, as well as live-cell microscopy. This new sensor responds to nanomolar and micromolar concentrations of ADP and ATP in solution, respectively, and in principle it is the first fully-genetically encoded sensor with sufficiently high affinity for ADP to detect low levels of extracellular ADP. Furthermore, we demonstrate that tethering the sensor to the cell surface enables the detection of physiologically relevant nucleotide release induced by hypoosmotic shock as a model of tissue edema. Thus, we provide a new tool to study purinergic signaling that can be used across genetically tractable model systems.

## 1. Introduction

Purinergic signaling regulates a broad range of critical physiological processes such as neuron-glia communication, immune cell responses, and platelet aggregation, and may be a key determinant of disease states such as cancer [1,2,3,4,5,6]. In injuries that result in tissue edema, purinergic signaling also plays an important role in the cell volume regulatory response to changes in osmotic pressure [1,6,7,8,9,10,11,12]. In purinergic signaling, extracellular adenosine triphosphate (ATP) is a ligand of P2X ion channels and several P2Y G-protein coupled receptors, and extracellular adenosine diphosphate (ADP) can also activate several P2Y receptors [1,2,3,4,5,6]. As such, there is a need for sensitive detection methods to measure the release and clearance of extracellular nucleotides such as ATP and ADP, as well as adenosine in order to understand the stress response. We recently developed a genetically-encoded fluorescent sensor of extracellular ATP [13], but there has not been a genetically encoded fluorescent sensor of ADP until now. One major challenge to the development of these purinergic sensors is that extracellular nucleotides signal at low concentrations, putatively at hundreds of nanomolar to low micromolar levels [14]. Here, we take advantage of a bacterial protein that has a high affinity for ADP, called ParM, and use it to engineer an extracellular ADP sensor successfully.

**Figure 1:**
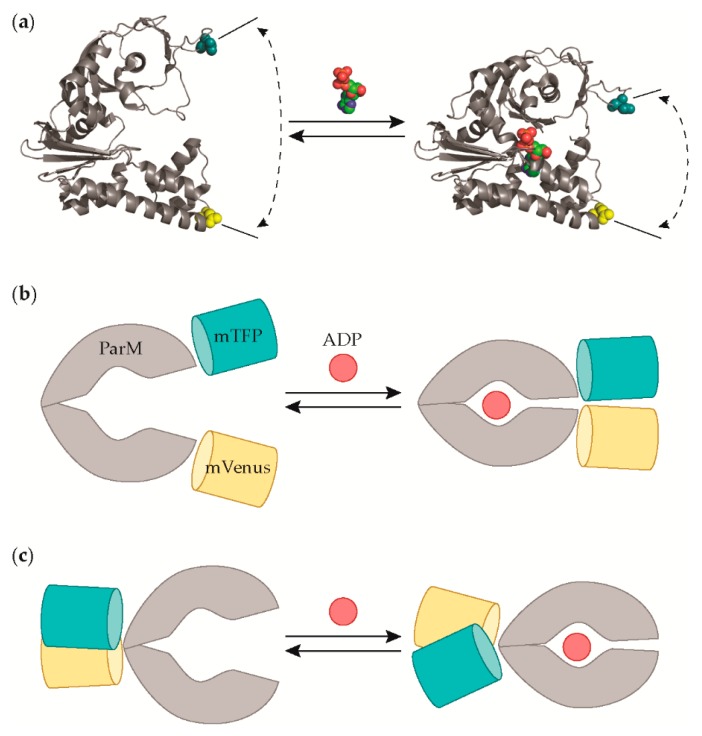
Adenosine diphosphate (ADP) sensor designs. (**a**) The X-ray crystal structures of *E. coli* ParM in the apo (PDB 1MWK) and ADP-bound (1MWM) states illustrate the ADP-dependent conformational change. (**b**) In the “insertion-fusion” sensor design, the cyan (mTFP) and yellow (mVenus) fluorescent proteins are inserted within the ParM protein at the apical surface-exposed loops, which are indicated in (**a**) by the residues shown as cyan and yellow spheres. A decrease in donor-acceptor distance upon ADP-binding would be expected to increase FRET. (**c**) In the “terminal-fusion” sensor design, the fluorescent proteins are fused to the N- and C-termini of ParM. The termini are in close proximity to one another, and therefore, an ADP-dependent change in the relative orientation between the donor and acceptor would be expected to be the primary cause for a change in FRET.

ParM is an actin-like *Escherichia coli (E. coli)* protein that exhibits a large conformational change upon binding ADP (Figure 1) [15]. Previously, Kunzelmann and Webb demonstrated that ParM could be used to generate a semisynthetic ADP sensor through a structure-guided cysteine mutagenesis and dye conjugation strategy [16,17]. Their work provides excellent proof that ParM is a fruitful scaffold for rational sensor design. However, because we are ultimately interested in developing an extracellular ADP sensor capable of cell type-specific surface display, we asked whether ParM could also be used to engineer a fluorescent protein-based genetically encoded sensor. Despite the clear precedent established by Kunzelmann and Webb, the dramatic size difference between fluorescent proteins and the small organic dyes used previously [16,17] left the answer to this question uncertain. Here, we now report the successful development of a genetically encoded ParM-based sensor that reports fluctuations in physiological levels of extracellular ADP through changes in Förster-type resonance energy transfer (FRET) between a cyan fluorescent protein (CFP) and a yellow fluorescent protein (YFP).

## 2. Materials and Methods

Chemicals. Unless otherwise noted, all chemicals and cell culture reagents were from Sigma-Aldrich and Thermo Fisher Scientific. Synthetic gBlock DNA and oligonucleotides were from Integrated DNA Technologies.

Protein Engineering and Library Screening. The *E. coli* ParM gene was synthesized as a gBlock and subcloned into the pRSETB bacterial expression vector. The genes for the mTFP1 and mVenus wildtype and circularly permuted variants were subcloned from the Pertz Lab cpFRET biosensors kit (Addgene kit # 1000000021) [18]. The fusions between ParM and the fluorescent proteins were generated using the NotI and BspEI restriction enzyme sites engineered in the Pertz kit gene constructs, using PCR mutagenesis to create appropriate restriction sites in the ParM gene. Standard restriction enzyme, PCR mutagenesis, and Gibson Assembly (NEB HiFi Kit) approaches were used to generate linker libraries.

Individual plasmids or plasmid libraries were transformed into DH5α *E. coli* and grown in Luria Broth media shaking at 37 °C overnight followed by two additional days of shaking at ambient temperature. Leaky expression was sufficient for library screening and protein purification. For library screening, 96-well deep-well plates were inoculated with a single colony per well and grown as described. Bacterial cultures were then washed and diluted to an OD_600_ of 0.2 with minimal M9 media (50 mM Na_2_HPO_4_, 20 mM KH_2_PO_4_, 20 mM NH_4_Cl, 9 mM NaCl, 2 mM MgSO_4_, 0.1 mM CaCl_2_, pH 7.5). Steady-state fluorescence emission spectra were measured on a BioTek Synergy H4 Multimode Plate Reader using 450 nm excitation with a 9 nm bandwidth. The emission ratio was calculated as the mTFP1 peak emission band intensity at 495 nm divided by the mVenus sensitized FRET emission intensity at 525 nm, both with 9 nm bandwidths. After the baseline spectra were measured, KCN was added to a final concentration of 10 mM [19], and cells were incubated for 10 min to allow for the depletion of ATP and accumulation of ADP before a second set of spectra were measured.

For time-resolved spectroscopy, fluorescence lifetime decays were measured using time-correlated single-photon counting on an Edinburgh Instrument FS5+ spectrometer with a NKT Photonics Fianium WhiteLaseMicro pulsed laser source. Live *E. coli* suspensions were prepared as described above except that cultures were diluted to an OD_600_ of ~0.5 and pre-starved for 30–60 min in the absence of glucose. Live-cell lifetime decays were collected using Fluoracle software, and empirical lifetimes were measured and analyzed in Matlab as previously described [20].

In vitro characterization. Sensor protein was expressed in DH5α *E. coli*, grown in Luria Broth media shaking at 37 °C overnight followed by two additional days of shaking at ambient temperature. Cells were lysed and purified using nickel affinity chromatography on a HiTrapTM Chelating HP column. The purified protein was dialyzed to remove excess concentrations of imidazole. The dose-response assays were performed with a BioTek Synergy H4 Multimode Plate Reader at a protein concentration of 0.125 µM in assay buffer (50 mM MOPS-KOH, 50 mM KCl, 0.5 mM MgCl_2_, 0.05% Triton-X, pH 7.3). ADP and ATP were complexed with magnesium prior to use in the dose-response assays. Nucleotide concentration was confirmed via absorbance measurements. The Origin software package was used to fit the dose-response data and calculate the K_d_ using the Hill function.

Live-cell microscopy. The sensor gene used for soluble expression was subcloned into the pCMV(MinDis) mammalian cell-surface expression vector, in which the sensor was tethered to the outer face of the plasma membrane by the platelet-derived growth factor receptor (PDGFR) transmembrane domain and an Igκ secretion signal, obtained from the iGluSnFR developed by the Looger Group [21]. HEK293A cells were plated on nitric acid cleaned glass coverslips and transfected by the calcium phosphate method. The coverslips were placed in a Warner superfusion chamber with a continuous flow of imaging solution (120 mM NaCl, 15 mM HEPES, 10 mM glucose, 3 mM KCl, 3 mM NaHCO_3_, 2 mM CaCl_2_, 1.25 mM NaH_2_PO_4_, 1 mM MgCl_2_, pH 7.3) at ~2 mL/min at ambient temperature. The cells were equilibrated in the chamber for a minimum of 15 min prior to imaging. For live-cell dose-response experiments, ADP and ATP were diluted in imaging solution to the appropriate final concentration. For hypoosmotic shock, an imaging solution containing a reduced concentration of 60 mM NaCl was used. Images were collected on an Olympus IX83 microscope with an Andor Zyla 4.2 sCMOS camera set at 2-by-2 pixel binning and 100 millisecond-exposure times and using 20X 0.75 NA PlanApo air objective. Images were collected at a 10 s interval using a Lumencor SpectraX as an excitation source of 438 nm and 470 nm excitation and emission filters to collect CFP donor fluorescence, 438 nm and 540 nm filters to collect CFP-YFP FRET emission, and 510 nm and 540 nm filters to collect YFP acceptor fluorescence, respectively. NIH ImageJ software was used for ratiometric image analysis. Binary masks were generated by using a minimum threshold of the mean plus three standard deviations of the background intensity. The binary mask was applied to the background-subtracted images for each channel, and a CFP/FRET pixel-by-pixel ratio image was calculated. The membrane regions of interest (ROIs) were manually drawn for each cell, and the ratio changes over time were normalized to a linear baseline.

## 3. Results

Our objective was to engineer a high affinity genetically encoded sensor of extracellular ADP by fusing a CFP and YFP FRET pair to the *E. coli* ParM protein. To do so, we first developed a live-cell assay to screen soluble candidate sensor libraries. After screening several libraries, we identified a viable sensor and characterized its performance in solution studies. Finally, we then generated a sensor construct targeted to the cell surface and validated that this new sensor detects the physiologically relevant release of extracellular nucleotides.

### 3.1. Protein Engineering and Library Screening

In our initial designs of the sensor, we explored two possible FRET-based architectures that differed in the positioning of the CFP-YFP FRET pair when fused to the ParM protein in order to take advantage of alternative aspects of the ADP-dependent structural change. Upon binding ADP, the ParM protein undergoes a hinge-like conformational change in which the two domains move closer to one another (Figure 1) [15]. In our first design, we sought to primarily exploit the distance-dependence of FRET by fusing the CFP and YFP to opposing sites on the two different ParM domains (Figure 1). Previously, Kunzelmann and Webb conjugated a coumarin dye to the I27 cysteine mutation site that experiences a change in solvent exposure upon ADP binding [17], as well as rhodamine dyes to the D63 and D224 cysteine mutation sites that come in close enough proximity to enable dye-dye interactions [16]. However, we sought alternative sites to fuse the CFP and YFP because we were concerned that the ParM structure would not accommodate the large bulk of a fluorescent protein, especially at the I27 and D224 sites lining the ADP binding site [15]. Instead, we selected CFP and YFP insertion sites at D41 and D241 because the distance between these two residues decreases by nearly 20 Å upon ADP binding, and they are found on apical surface loops on each respective domain, which we hypothesized would better tolerate the fusion of large fluorescent proteins (Figure 1).

In our second design, we directly fused the CFP and YFP to the N- and C-termini of the ParM protein (Figure 1). Although the ParM termini are located close to one another in spatial proximity, previous work developing a glucose sensor has demonstrated that this is a viable strategy for generating FRET sensors [22]. In this strategy, we sought to exploit the sensitivity of FRET to the orientation between the donor and acceptor, hypothesizing that the allostery of ParM would be transduced into a change in the relative dipole-dipole angle between the CFP and YFP fused to the termini.

For both sensor designs we used ParM harboring select mutations that include: the K33A mutation which prevents ParM polymerization; the T174A and T175N mutations which reduce ATP affinity and increase ADP selectivity; and the C287A mutation to remove a surface-exposed cysteine as a conservative precaution against unwanted redox reactions with the protein [15,16,17]. The K33A mutation was also demonstrated to abrogate the ATP hydrolysis activity in two different constructs [16,17].

**Figure 2:**
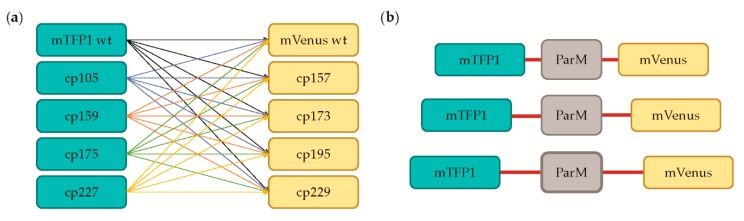
Diagram of ADP sensor libraries. (**a**) Different relative orientations between the mTFP1 and mVenus FRET pair was achieved through the circular permutation kit developed by Pertz and co-workers [18]; (**b**) In addition, different linker lengths and linker compositions were screened.

FRET is dependent on both distance and relative orientation between the CFP donor and YFP acceptor; therefore, we generated libraries for both the “insertion-fusion” and the “terminal-fusion” designs using the “cpFRET” biosensor construction kit developed by the Pertz Lab [18]. This kit utilizes the cyan mTFP1 [23] as the donor and the yellow mVenus as the acceptor [24]. In addition to the wildtype versions of these fluorescent proteins, Pertz and co-workers constructed four circular permutation variants of each, in which the N- and C-termini are relocated to different locations in the β-barrel structure [18]. For example, in the mTFP1-cp105 circular permutation variant, the native termini have been fused with a short peptide linker, and new termini are generated at residue 105. Because of the different termini locations, these circular permutation variants enabled us to generate combinatorial libraries to screen for an mTFP1-mVenus pair with the optimal orientation for an ADP-dependent FRET change when fused to ParM (Figure 2a).

**Figure 3:**
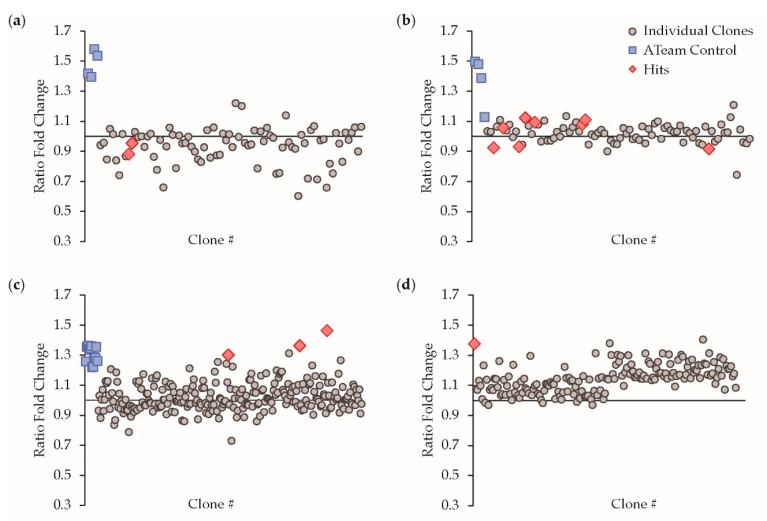
Library screening results. Results are shown for the (**a**) insertion-fusion, (**b**) terminal-fusion, (**c**) linker length, and (**d**) polyproline linker composition library screens. The y-axis values represent the fold change in the mTFP1/mVenus FRET ratio after KCN addition relative to baseline measurements for live-cell suspensions. Each symbol represents the result for a culture grown from a single clone in the library, and the responses of individual clones are plotted along the x-axis according to Clone #. The blue squares represent the response of the ATeam1.03 ATP sensor as a control. The red diamonds represent “hits” in which a response was both ratiometric and could be reproduced, except in (**d**) where the red diamond represents the response of the best hit from the library in (**c**) as a control.

In order to screen the cpFRET insertion-fusion and terminal-fusion libraries, sensor candidates were expressed and directly assayed in live *E. coli*. In the assay, single clones were inoculated in liquid cultures in a multi-well plate format, and subjected to metabolic inhibition with cyanide. Studies with fluorescent ATP sensors have demonstrated that cyanide treatment causes a decrease in bacterial ATP levels [19]. We validated that cyanide caused a decrease in ATP in our assay using the ATeam1.03 ATP sensor [25], which we also used as an assay control in our screens (Figure 3). Furthermore, we used a luciferase-based biochemical assay to confirm that cyanide treatment causes a four-fold increase in bacterial ADP levels (Figure S1), thus providing the basis for our screen. To screen our libraries, steady-state fluorescence emission spectra were taken on the live-cell suspensions before and after cyanide treatment. As our selection criteria, candidate sensor hits were chosen if they exhibited peaks for both the donor mTFP1 fluorescence and sensitized emission from mVenus, as well as a ratiometric change in the fluorescence emission spectra after metabolic inhibition. Note that throughout this manuscript, we use the “C/Y FRET Ratio” equal to the mTFP1 donor emission intensity divided by the mVenus-sensitized acceptor FRET emission intensity because the final selected sensor exhibits a decrease in FRET upon ADP binding, which is a convention used for other FRET sensors [26]. In Figure 3, we quantify the spectral changes as the “Fold Ratio Change” equal to the C/Y FRET Ratio before treatment divided by the C/Y FRET Ratio after metabolic inhibition. Thus, a Fold Ratio Change greater than one indicates a decrease in FRET upon ADP binding, whereas a Fold Ratio Change less than one indicates an increase in FRET upon ADP binding. For example, the control ATeam1.03 ATP sensor exhibits an increase in FRET upon ATP binding. Hence, metabolic inhibition causes a decrease in ATP and a decrease in ATeam1.03 FRET, and control samples in Figure 3 (blue squares) exhibit a Ratio Fold Change greater than one.

Despite the large ADP-dependent decrease in distance between insertion sites, the insertion-fusion library only yielded two candidate hits (Figure 3a). Several of the other clones exhibited large fluorescence changes but were rejected as false positives because their responses did not meet our aforementioned criteria, such as a lack of a clear ratiometric spectral change upon closer inspection. In contrast, eight candidate hits were selected from the terminal-fusion library that exhibited ratiometric responses. Interestingly, of the total ten candidate hits from our library screens, there was a diversity of wildtype and circularly permuted mTFP1 and mVenus pairings without a clear pattern (Table 1). There was also a diversity of ratiometric spectral changes in which some clones exhibited an increase in FRET upon metabolic inhibition (Ratio Fold Change < 1) while other clones exhibited a decrease in FRET upon metabolic inhibition (Ratio Fold Change > 1) (Figure 3).

**Figure 4:**
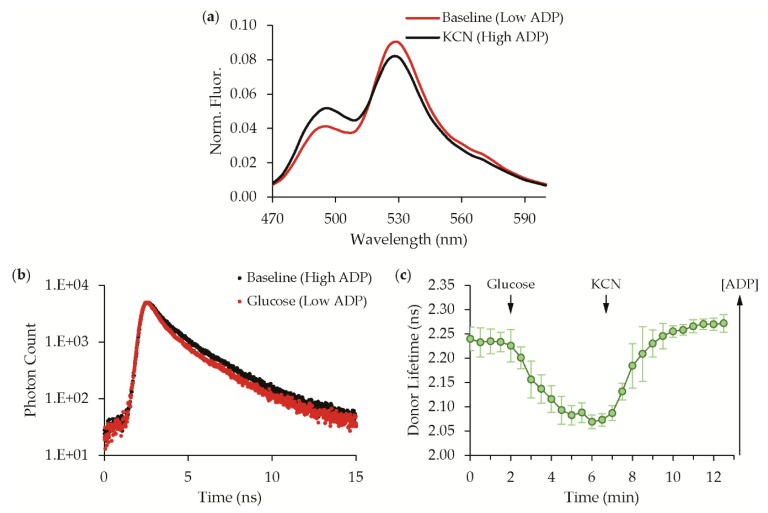
Live-cell assays validate the sensor response is reversible. (**a**) The steady-state fluorescence emission spectra (435 nm excitation) of the best sensor identified from library screening exhibits a ratiometric change in the mTFP1 donor emission peak and mVenus acceptor-sensitized emission peak. FRET decreases when ADP increases. The spectra are normalized to the total integrated fluorescence, and an isosbestic point at 515 nm is evident. In the live-cell assay, an increase in ADP is caused by metabolic inhibition of *E. coli* with cyanide (KCN). (**b**,**c**) When starved cells are fed glucose, the mTFP1 donor fluorescence lifetime decreases as expected because ADP is consumed. The sensor response is reversible, and the mTFP1 donor lifetime increases upon cyanide treatment, which causes a subsequent increase in ADP. Examples of raw fluorescence lifetime decays are shown in (**b**) and the time course of lifetime changes (mean ± 95%CI, n = 6) is shown in (**c**).

As the second stage of optimization, we next varied the length of the linkers that served to attach the fluorescent proteins to ParM (Figure 3c). We mutagenized the linkers to lengths of three, five, or seven amino acids in length on each end of both fluorescent proteins and carried out additional library screens. Again, we observed diverse responses to cyanide treatment, and we selected three top candidate hits for re-confirmation. To our surprise, upon sequencing these hits we discovered that all three represented the same sensor clone, providing unexpected internal validation for the selection of this sensor, and there was a clear ADP-dependent ratiometric change in its spectra (Figure 4a). This hit was the terminal-fusion construct that utilized the wildtype mTFP1 connected by a five amino acid length linker (SGITS) to the N-terminus of ParM, and the mVenus-cp157 variant connected by a three amino acid length linker (AAA) to the C-terminus of ParM. Here, the compositions of the linkers were dictated by the cloning strategy. Given that this sensor was derived from the terminal fusion library, we suspected that the primary cause for the FRET response is that ADP binding to ParM is transduced as a change in relative orientation between the two fluorescent proteins. As such, we carried out the third stage of optimization focused on the orientation of the fluorescent proteins in the sensor. Previously, Thestrup et al. screened polyproline linkers to vary and optimize the orientation of the fluorescent proteins in the Twitch calcium sensors [27]. We generated a similar polyproline linker composition library, but we did not identify any significantly improved variants compared to the hit from the linker length screen (Figure 3d). Therefore, we chose to carry out further characterization on our new ADP sensor.

### 3.2. Validation with Live-Cell Time-Resolved Spectroscopy

Upon addition of cyanide to live-cell cultures, the sensor reproducibly reports an increase in bacterial ADP levels with an increase in the donor mTFP1 emission peak and a reciprocal decrease in the mVenus sensitized emission peak (Figure 4a). To validate that the observed spectral change represents a change in FRET, we carried out time-resolved spectroscopy on live-cell cultures expressing the sensor [20]. From the spectral response, ADP-binding to the sensor causes a decrease in FRET, which should manifest as an increase in the mTFP1 donor fluorescence lifetime when ADP levels increase [28]. We also sought to validate that the sensor provides a reversible response, and therefore, in this assay, we pre-starved the *E. coli* in M9 minimal media without any amino acids or glucose to deplete ATP and increase ADP levels. At baseline in the starved state, the donor lifetime of the sensor is 2.24 ± 0.02 ns (mean ± 95% confidence interval (CI), n = 6). The addition of glucose to the cells causes consumption of ADP, which was reported by a decrease in the donor lifetime to 2.06 ± 0.02 ns (mean ± 95% CI, n = 6). The subsequent addition of cyanide caused a reversal, and the donor lifetime returned to a value of 2.27 ± 0.02 ns (mean ± 95% CI, n = 6) with the increased bacterial ADP levels. Thus, by using live-cell time-resolved spectroscopy, we validated that our newly identified sensor hit responds reversibly to fluctuations in ADP concentration with dynamic changes in FRET.

### 3.3. Protein Solution Characterization

**Figure 5:**
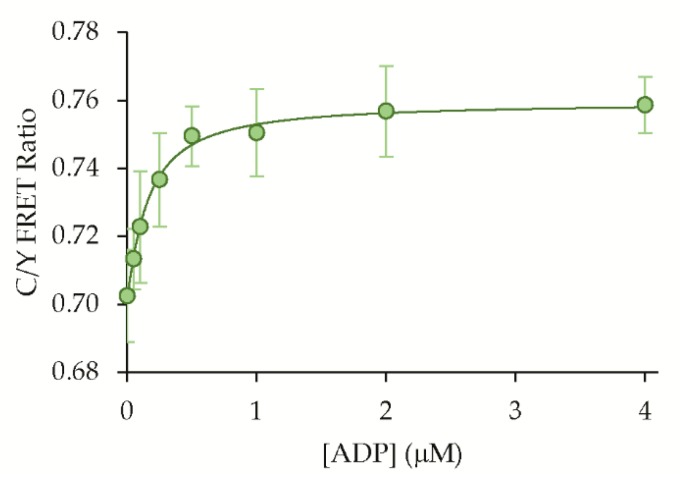
ADP affinity. The ADP dose response of the sensor was measured using purified protein in solution with an apparent K_d_ = 0.16 ± 0.06 µM (mean ± std, n = 9).

We next expressed and purified the ADP sensor in order to characterize its performance in solution studies. The ADP dose-response of the sensor showed a high affinity with an apparent K_d_ equal to 0.16 ± 0.06 μM (mean ± std, n = 9), which is similar to the previously reported value of ~0.5 μM for the core ParM protein (Figure 5) [17]. Notably, this high affinity is well-matched to the putative nanomolar to micromolar concentration range for extracellular nucleotides. We also observed a loss in the dynamic range compared to the live-cell assays, with a maximum FRET ratio change of 8%–10%. The difference in the dynamic range could be due to the difference in the crowded cellular environment versus dilute solutions, but the exact causes are unknown. We were encouraged, however, by the clear usefulness of other recent ratiometric FRET sensors with low dynamic ranges [26,29,30], and therefore, we proceeded with further characterization and proof-of-concept studies.

**Figure 6:**
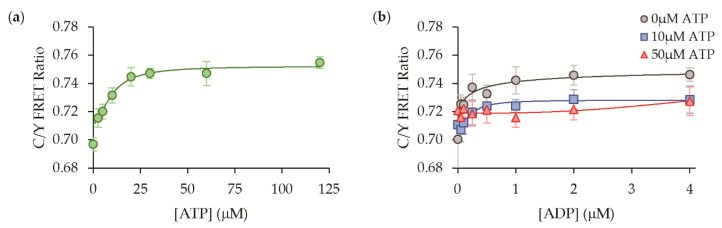
ATP binding and interference. (**a**) ATP elicits a fluorescence response with a significantly lower apparent affinity of K_d_ = 9.9 ± 6.3 μM in solution studies, maintaining a > 50-fold selectivity for ADP over ATP. (**b**) Increasing concentrations of ATP can attenuate the response of the sensor to ADP (10 mM ATP, K_d_ = 0.22 ± 0.09 mM; 50 mM ATP, no fit; mean ± std, n = 6).

The ParM protein, with its T174A and T175N mutations, has a reduced affinity for ATP as a competitive ligand, and we directly assessed the ATP binding to our sensor. The ATP dose-response revealed that the sensor exhibits a significantly lower affinity for ATP with an apparent K_d_ equal to 9.9 ± 6.3 μM (mean ± std, n = 3) (Figure 6a). Our sensor, thus, has a much higher affinity for ATP compared to the parent ParM protein (K_d_ ~200 μM [17]), but it still maintains a nearly 62-fold selectivity for ADP over ATP. We also found that the presence of ATP attenuated the sensor’s response to ADP. In the presence of 10 μM ATP, the sensor was still able to detect ADP with similar affinity but diminished the dynamic range (Figure 6b). At a saturating concentration of 50 μM ATP, the response to ADP was abolished. These interfering effects of ATP may in part qualitatively explain differences observed in the cell-based assays and dilute protein solution studies given that we measured a large change in ATP content upon cyanide treatment of the live bacterial cultures (Figure S1). However, tens of micromolar concentrations of ATP are thought to be pathologically high for basal fluctuations of extracellular nucleotides outside the cell. Thus, the sensor’s sensitivity, even in the presence of ATP, should be sufficient to detect changes in extracellular ADP.

**Figure 7:**
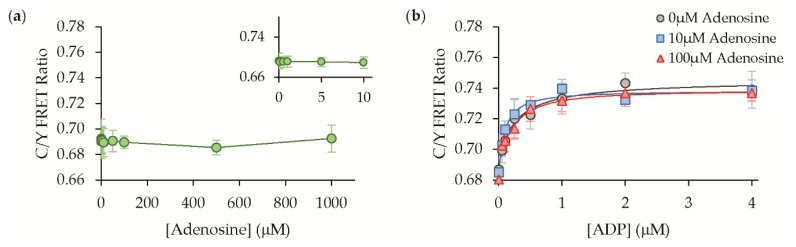
Adenosine binding and interference. (**a**) The sensor does not exhibit a direct response to adenosine, and (**b**) adenosine does not affect the sensor response to ADP in solution studies, mean ± std (n = 3).

Outside the cell, adenosine is another possible interfering molecule that could be encountered during purinergic signaling, but its binding to ParM has not been assessed previously [16,17]. We found that our ADP sensor exhibited no appreciable affinity for adenosine in its dose-response (Figure 7a). Furthermore, the presence of even very high concentrations of adenosine did not compromise the sensor’s response to ADP at all (Figure 7b).

**Figure 8:**
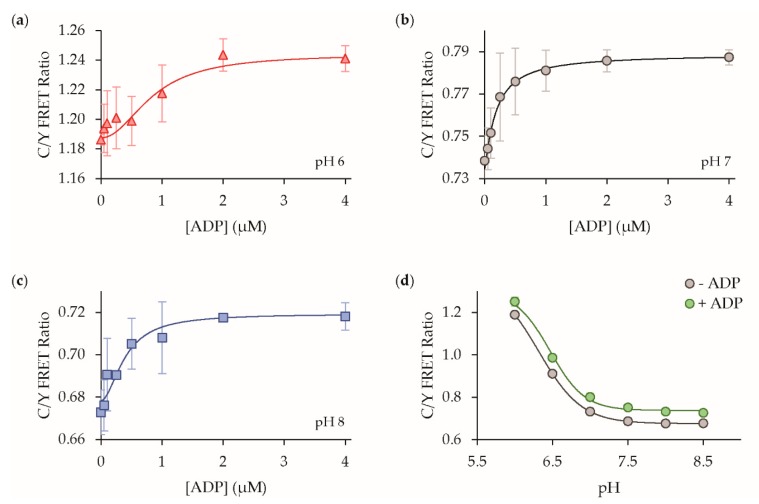
The pH sensitivity. ADP dose response curves at (**a**) pH 6 (K_d_ = 0.85 ± 0.29 mM); (**b**) pH 7 (K_d_ = 0.19 ± 0.07 mM); (**c**) and pH 8 in solution (K_d_ = 0.40 ± 0.10 mM); (**d**) The sensor is sensitive to changes in the absolute ratio values at pH 6.5 and below, but it is insensitive to pH changes above pH 7, mean ± std (n = 3).

Lastly in our solution characterization studies, we determined the pH sensitivity of our ADP sensor’s response. We found that the sensor’s ADP dose response is preserved with sub-micromolar affinity and similar dynamic range whether measured at pH 6, 7, or 8, but instead, there was a shift to larger absolute FRET ratio values at lower pH (Figure 8). Indeed, examining the pH dose-response, we found that FRET ratio exhibited very similar pK_a_ values of ~6.5 and ~6.3 in the absence or presence of ADP, respectively (Figure 8d). This pK_a_ value matches well with the pK_a_ of ~6 for mVenus [24], while mTFP1 is pH insensitive with a pK_a_ of 4.3 [23]. Given that the ADP affinity is not strongly perturbed by pH, these observations suggest that the pH sensitivity of the absolute FRET ratio signal is primarily due to the pH-sensitive mVenus and is not likely due to pH-sensitive nucleotide binding to ParM. In addition, we observed that the sensor response was stable at pH 7.0 and higher, which would be well-suited for many extracellular sensing studies in buffered bath solutions.

Although our protein solution studies revealed that our ADP sensor has a limited dynamic range, as mentioned, we were encouraged by the successful use of other low dynamic range FRET sensors in live-cell imaging studies [26,29,30]. In particular, ratiometric imaging could be quite robust and sensitive because of the intrinsic normalization against artifacts caused by varying expression levels or other heterogeneities, and thus, we next demonstrated proof-of-concept that our sensor could be used to detect extracellular ADP in live-cell cultures.

### 3.4. Cell Surface Expression and Characterization

**Figure 9:**
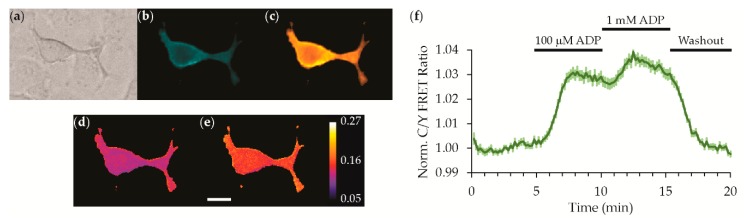
The ADPrime sensor responds to extracellular ADP. Widefield microscopy shows an example of a sensor expressing HEK293 cell. Panels show individual (**a**) differential interference contrast, (**b**) CFP, and (**c**) YFP channels as well as C/Y ratio images (**d**) before and (**e**) after ADP wash-in. The false color calibration bar is for the raw mTFP/FRET ratio. Scale bar = 50 μm. (**f**) Time course of ADPrime response on live cells, mean ± 95% CI (77 cells, 11 experiments).

In order to measure extracellular ADP at the cell surface, we genetically fused the sensor to a transmembrane anchor derived from the platelet-derived growth factor receptor with an IgK signal sequence [13]. For convenience, we named this cell surface-targeted construct “ADPrime” (ADP sensor for Ratiometric IMaging of Extracellular purines). It was clear, even with widefield microscopy, that ADPrime targeted well to the surface of live HEK293 cells, which showed strong fluorescence signals at the membrane. To establish that ADPrime responds to extracellular ADP, we subjected the sensor expressing HEK293 cells to continuous superfusion and washed in a high, near-saturating concentration of 100 μM ADP, to ensure we would see a visible response. On average, we observed a dampened response compared to the soluble protein with a 3% increase in FRET ratio, and subsequent increase to 1 mM ADP by wash-in only increased the FRET ratio to 4% indicating saturation as expected (Figure 9). The response was fully reversed upon washout with an imaging solution lacking any ADP. In other studies, we did not observe robust responses to 1 mM ADP, but we did observe saturation of the response at 10 mM ADP (Figure S2), indicating that ADPrime exhibits micromolar apparent affinity when expressed on the surface of live cells. This attenuation in response and decrease in affinity compared to the sensor’s solution behavior is not entirely unexpected because we observed a very similar attenuation in the response and affinity of the ATeam sensor when we previously developed a cell surface-displayed extracellular ATP sensor [13]. We also note that the response to ADP wash in and wash out had time constants of ~60 s because the apparent on and off-rate kinetics are limited by our perfusion and mixing system. Furthermore, expression of ADPrime on the cell surface did not have any overt negative effects on cell viability (Figure S3). Despite the relatively small change in FRET ratio, the maximum response of 4.4 ± 0.2% (mean ± 95% CI) was highly reproducible across 77 cells in 11 independent experiments. Given the promising result that ADPrime provides in response to the exogenous addition of extracellular ADP, we next tested if it could detect a physiological release of ADP.

### 3.5. Detection of Physiological Nucleotide Release

**Figure 10:**
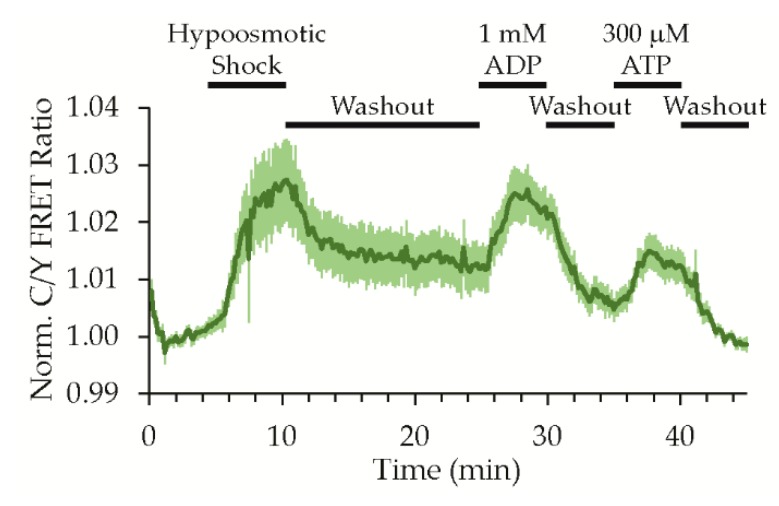
ADPrime detects hypoosmotic shock-induced nucleotide release. Time-course response of ADPrime expressed on live HEK293 cells with continuous superfusion and wash-in of solutions as indicated (mean ± 95% CI, 62 cells, nine experiments).

To demonstrate that ADPrime could detect a physiologically relevant nucleotide release, we subjected sensor expressing HEK293 cells to hypoosmotic stress because purinergic signaling is well established to play an important role in the volume regulatory response in a broad number of cell types [1,7,8,9,10,11,12]. After measuring a baseline with continuous superfusion of an isotonic imaging solution (~300 mosmol), hypotonic imaging solution (~150 mosmol) was washed in. The hypotonic solution was reduced by half in sodium chloride concentration but still contained the other buffer and salt components. This reduction in osmolality caused an immediate increase in the ADPrime FRET ratio to a maximum response of 3.9 ± 0.8% (mean ± 95% CI, 62 cells, nine experiments), reporting a release of extracellular nucleotides consistent with previous reports using similar in vitro models (Figure 10) [7,8,9,10,11]. The ADPrime response could be reversed by washout and return to the isotonic solution, and we validated that the sensor remained responsive with a subsequent pulse of exogenous ADP. Nucleotide release can involve the direct secretion of ATP and ADP to the extracellular space, and extracellular ATP is also quickly converted to ADP by ectonucleotidases such as CD39 [6]. We found that the sensor also responded to a pulse of exogenous ATP, as expected from our characterization of the selectivity in protein solution studies. Thus, ADPrime can reproducibly detect a hypoosmotic shock-induced release of purines, though it is not clear if it is detecting ADP, ATP, or both in this particular experiment. In this study, we focus on our first generation ADPrime sensor, but in future studies beyond the scope of this work, ADPrime could be combined with ecATeam [13] to distinguish between ADP and ATP species. Regardless, both ADP and ATP are involved in purinergic signaling, indicating that ADPrime could be used to monitor signaling dynamics.

## 4. Discussion

In this study, we developed a genetically encoded fluorescent sensor of extracellular ADP called ADPrime and demonstrated that it detects the release of extracellular nucleotides from live cells.

One of our major objectives was to show that the *E. coli* ParM protein could be combined with fluorescent protein technology to engineer this sensor, building on the work of Kunzelman and Webb [16,17]. Previously, Kunzelman and Webb used a semisynthetic strategy by conjugating synthetic dyes to cysteine-mutants of ParM. Their strategy yielded two sensors that exhibit three-fold and 10-fold maximal responses to ADP binding, and the high dynamic ranges result from the unquenching of the small organic fluorophores used, making these sensors ideal for solution studies. Clearly, ADPrime offers a significantly reduced dynamic range compared to these semisynthetic sensors, which is typical of ratiometric FRET sensors compared to intensity-based turn-on fluorescence sensors. However, FRET sensors with low dynamic range have still proven to be of great use for live-cell imaging studies because they are ratiometric, which provides an intrinsically normalized signal that improves quantitative comparisons across independent trials, and because they are genetically targetable. For example, the TORCAR FRET sensor exhibited a 13% FRET dynamic range and was effectively used to study the subcellular distribution of mTORC activity in live cells [26]. In addition, the Laconic [29] and Pyronic [30] FRET sensors of lactate and pyruvate exhibit 11% and 24% FRET dynamic ranges, respectively. These sensors have been used successfully to study brain energy metabolism in cultured cells, brain slices, and in vivo [31,32]. Similarly, we carried out a physiological proof-of-concept and demonstrated that ADPrime was effective in detecting the hypoosmotic stress-induced release of ADP and ATP in live-cell imaging studies. In particular, because ADPrime is entirely genetically encoded, it could be tethered to the cell exterior without the need for any surface chemistry. Thus, our sensor shows that ParM provides a high-affinity scaffold that is appropriate for the detection of the low concentrations of extracellular ADP that are involved in purinergic signaling.

As a first-generation extracellular ADP sensor, ADPrime was already able to detect endogenous purinergic signals. However, we were unable to discriminate between extracellular ATP and ADP with certainty in our hypoosmotic shock studies despite the 62-fold selectivity for ADP or ATP. It is not clear why ADPrime exhibits a decrease in nucleotide selectivity compared to other ParM constructs that show greater selectivity for ADP over ATP. It is possible that steric constraints imposed by the fusion to the large fluorescent proteins alter the coupled equilibrium between nucleotide binding and the closed and open states. In future studies during the development of next-generation sensors, a mutational analysis may shed light on this interesting observation. Regardless of the needed improvements in selectivity, ADPrime is the first fully genetically encoded sensor that harbors a sufficiently high affinity for ADP that matches extracellular levels expected during purinergic signaling.

ADPrime could also be improved by increasing the FRET dynamic range. In particular, we measured that the mTFP1 donor lifetime in ADPrime is ~1 ns shorter than 3.2 ns lifetime of mTFP1 alone [23], suggesting that ADPrime experiences relatively high FRET in both apo and ADP-bound states. Thus, it may be fruitful to optimize the change in orientations of the FRET pair through further optimization of the fluorescent protein linkers [27] and by testing new fluorescent proteins that have been reported to serve well as high-efficiency FRET pairs [33]. Furthermore, in this study, we chose a FRET strategy because of the extensive number of examples using this strategy to re-purpose conformationally dynamic ligand-binding domains into sensors and because ratiometric sensors intrinsically normalize for expression level when considering live-cell studies [34]. However, we demonstrated that the ParM scaffold tolerates fusions to fluorescent proteins, and therefore a number of other sensor architectures, such as those using circularly permuted fluorescent proteins, could be explored in the future.

Interestingly, we also observed that ADPrime showed a ~0.2 ns change in mTFP1 donor fluorescence lifetime, which is comparable to other FRET probes, such as recent iterations of the ERK and PKA sensors that show 0.20–0.25 ns lifetime changes and have been used to study neuronal signaling dynamics [35]. Although fluorescence lifetime imaging microscopy (FLIM) is beyond the scope of this study, ADPrime has a lifetime dynamic range that could make it appropriate for live-cell FLIM studies.

Overall, ADPrime is an important new addition to the purinergic signaling toolkit because sensors of extracellular ADP had not been available until now. In combination with other sensors such as ecATeam [13], jrGECO1a [36], and PinkFlamindo [37], for example, ADPrime will be useful for quantitatively studying both upstream purinergic signal release dynamics and downstream G-protein Ca^2+^ and cAMP signaling.

## Figures and Tables

**Table 1:** The fluorescent protein variants for example library screen “hits”.

Hit ID	mTFP1 Variant	mVenus Variant
Insertion Clone 1	WT	cp157
Insertion Clone 2	cp227	cp157
Terminal Clone 1	cp105	cp173
Terminal Clone 2	WT	cp157
Terminal Clone 3	WT	cp173
Terminal Clone 4	cp159	WT
Terminal Clone 5	WT	cp195
Terminal Clone 6	cp227	WT
Terminal Clone 7	cp227	cp195
Terminal Clone 8	cp227	cp173

## Supplementary Materials

The following are available online at https://www.mdpi.com/1424-8220/19/15/3253/s1, Figure S1: Luciferase-Based ATP and ADP Measurements; Figure S2: Live-cell response to 10 μM ADP; Figure S3. Cell growth and death of transfected cells.
