# Supplementary material for: Detection of Osmotic Shock-Induced Extracellular Nucleotide Release with a Genetically Encoded Fluorescent Sensor of ADP and ATP
Source: Sensors (Basel). 2019 Jul 24;19(15):3253. doi: 10.3390/s19153253 (PMC6695626; doi:10.3390/s19153253)
Supplement: Supplementary file 1 [file sensors-19-03253-s001.pdf]

Supplementary Material

Detection of Osmotic Shock-Induced Extracellular Nucleotide Release with a Genetically Encoded Fluorescent Sensor of ADP and ATP

Keelan J. Trull [1], Piper Miller [1], Kiet Tat [1], S. Ashley Varney [1], Jason M. Conley [2] and Mathew Tantama [1,3,4,*]

[1] Department of Chemistry, Purdue University, 560 Oval Drive, West Lafayette, IN 47907, USA
[2] Department of Pediatrics, Indiana University School of Medicine, Indianapolis, IN 46202, USA
[3] Purdue Institute for Integrative Neuroscience, Hall for Discovery Learning #399, 207 South Martin Jischke Drive, West Lafayette, IN 47907, USA
[4] Department of Chemistry, Wellesley College, 106 Central Street, Wellesley, MA 02481, USA
* Correspondence: mt4@wellesley.edu

CONTENTS

Supplementary Methods
Figure S1. Luciferase-Based ATP and ADP Measurements
Figure S2. Live-cell response to 10 μM ADP
Figure S3. Cell growth and death of transfected cells

Supplementary Methods

Luciferase Assay. DH5α E. coli were grown as described for library screening. Briefly, cultures were diluted to an OD of 0.20 and treated with 10mM KCN or vehicle for 10 min. Then, 300 μL samples of the cell suspensions were transferred to separate wells of a 96-well plate on ice. Cell pellets were collected by centrifugation and supernatants discarded. Cells were lysed by the addition of 30 μL of pre-chilled 1X PCA Lysis Solution (500 mM perchloric acid, 2 mM EDTA) with shaking at 4 °C for 5 min. Then, cells were subject to a single round of freeze thaw. Perchloric acid was neutralized and precipitated with 30 μL of pre-chilled 0.5 M KOH with shaking at 4 °C for 5 min plus an additional 5 min incubation on ice. Debris was pelleted by centrifugation for 5 min × 1000 g in a swinging bucket rotor, then cleared lysate samples held on ice. Prior to measurement using the Perkin Elmer ATPLite kit, cleared lysate samples and nucleotide standards were first diluted. Duplicate dilutions from each cleared lysate sample were made for ATP and ADP measurements, respectively, by diluting 50 μL of cleared lysate with an additional 100 μL 1.5X Dilution Buffer (112.5mM Tris-HCL pH 8, 73 mM KCl, 7.5 mM $MgCl_2$, 0.15 mg/mL BSA) in separate wells. ATP and ADP standard curves were made by adding 10 μL of ATP and ADP serial dilutions in separate wells containing 50 μL of 1.5X Dilution Buffer. Samples were shaken for 1 min at 4°C to mix well. For cleared lysate samples and ADP standards, 10 μL of PK/PEP Solution (1 U/μL pyruvate kinase, 500 mM phosphopyruvate) was added to convert ADP to ATP. Finally, 60 μL diluted cleared lysate samples or diluted nucleotide standards were transferred to new wells of 96-well plate, and ATP content was measured using the ATPLite kit according to the manufacturer's instructions under dimmed lights with modifications. Briefly, 20 μL of ATPLite Lysis solution was added to every well and immediately 20 uL of ATPLite Substrate Solution was added. Samples were shaken at ~700 rpm at RT for 5 min to mix well, and then luminescence reading were measured in kinetic mode of a BioTek Synergy H4 Multimode plate reader for 60–120 min to observe steady-state (~1 s integration, PMT sensitivity ~200).

Cell Growth/Death Assay. HEK293A cells were plated in a 24-well plate at a seeding density of 50,000 cells/well. One day after seeding, cells were transfected with 800ng of DNA (100% ADPrime

DNA, 50% ADPrime DNA and 50% carrier salmon sperm DNA, or 100% carrier salmon sperm DNA) via a calcium phosphate transfection. At one day post transfection the entire 500 µL volume of growth media containing detached cells was collected, adherent cells were washed then collected with 100 µL of trypsin, and adherent and detached cells were combined in the collected media. Cells were pelleted at 500xg for 6 min, supernatant was removed, and cells were resuspended in 50 µL of DPBS. 10 µL of cell suspension was combined with 10 µL of trypan blue and counted using a hemocytometer. The process was repeated at two days post transfection. Cell viability was calculated as the fraction of live cells to total cell count.

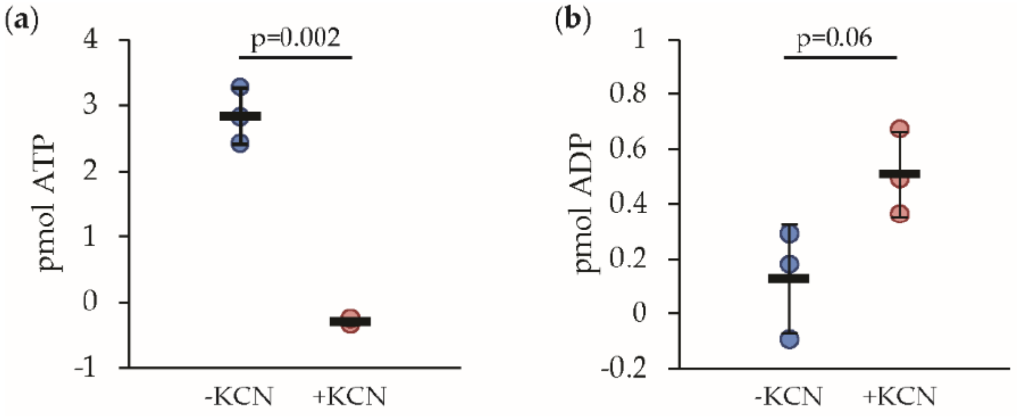

Figure S1. Luciferase-based ATP and ADP measurements. Live *E. coli* suspensions were treated with vehicle or with potassium cyanide, and then a sample was taken for a luciferase-based biochemical measurements that show cyanide causes (**a**) a decrease in ATP and (**b**) increase ADP content.

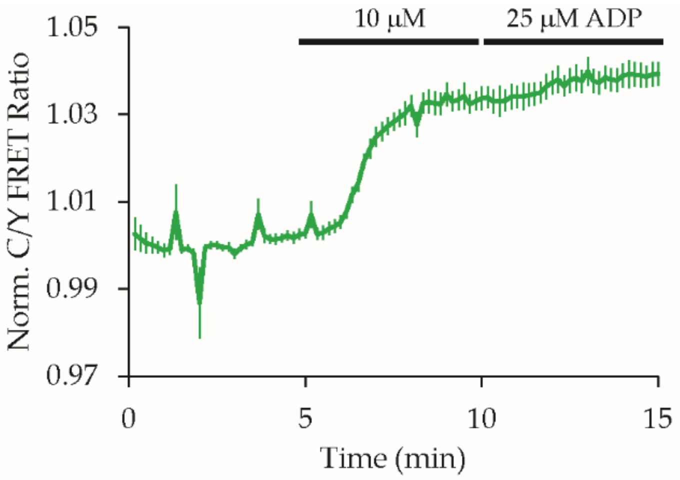

Figure S2. Live-cell response to 10 µM ADP. The ADPrime sensor response saturates after the addition of 10 µM ADP, and the ratio signal does not increase significantly upon wash-in of 25 µM ADP. mean ± 95%CI for 32 cells across 4 independent cultures.

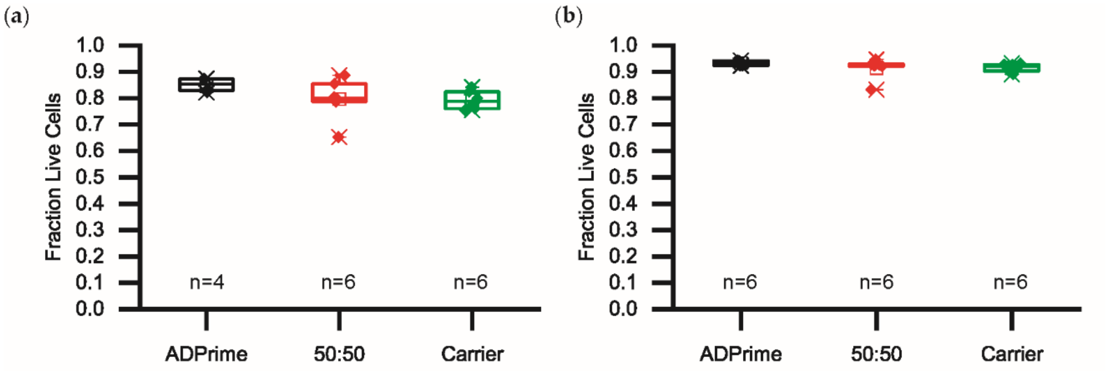

Figure S3. Cell growth and death of transfected cells. Expression of ADPrime does not affect cell viability compared to transfection controls. HEK293 cells were transfected with 800 ng of total DNA consisting of ADPrime plasmid DNA only, a 50:50 mix of ADPrime DNA and salmon sperm carrier DNA, or salmon sperm carrier DNA only. Detached cells in the media and adherent cells released by trypsinization were pooled, and viability was measured after (**a**) 1 day post-transfection and (**b**) 2 days post-transfection by trypan blue exclusion. The faction of live cells is the ratio of live cells to total cell count. One-way ANOVA with Tukey's post-hoc test and Levene's test on absolute deviations found no significant differences for the means or the variances.
